# Non-Articular Felty Syndrome Refractory to Granulocyte Colony-Stimulating Factor Therapy

**DOI:** 10.7759/cureus.17206

**Published:** 2021-08-15

**Authors:** Hasham Saeed, Chidinma Ejikeme, Marina Tucktuck, Qirat Jawed, William Kessler

**Affiliations:** 1 Internal Medicine, Rutgers Health/Trinitas Regional Medical Center, Elizabeth, USA; 2 Internal Medicine, St. George's University Medical School, Elizabeth, USA; 3 Hematology and Medical Oncology, Rutgers Health/Trinitas Regional Medical Center, Elizabeth, USA

**Keywords:** felty syndrome, rheumatoid arthritis, gsf refractory leukopenia, neutropenia, non-articular felty syndrome

## Abstract

Felty syndrome (FS), an uncommon manifestation seen in patients with rheumatoid arthritis (RA), usually presents as a triad of erosive arthritis, splenomegaly, and neutropenia. It is extremely rare for RA to present as FS or develop after initially presenting as neutropenia and splenomegaly. In this report, we describe a case of a 55-year-old woman who initially presented with fever and vaginal pain. Her sepsis workup revealed genital herpes in the setting of leukopenia, with an incidental finding of splenomegaly on imaging. The patient was managed with filgrastim and valacyclovir. Two weeks later, she presented again with pleuritic chest pain and worsening leukopenia. This led to an extensive workup by the hematology team to diagnose and confirm the diagnosis of FS. We also engage in a review of the existing literature of such cases and emphasize the importance of serological testing for RA in patients with leukopenia and splenomegaly, even in the absence of joint symptoms or prior diagnosis of RA. The management should be guided towards treating the infection, correcting the neutropenia, and treating the underlying chronic disease.

## Introduction

Felty syndrome (FS) is a rare complication of rheumatoid arthritis (RA), which is seen in patients with seropositive RA. The lifelong prevalence of FS is approximately 1-3% [1]; women account for 60-80% of the total patient population, with an average age of onset in the late 30s-early 40s.

Patients who present with FS usually have deforming arthritis due to years of suffering from RA. However, this is not always the case as there have been a few case reports of FS presenting in patients without clinical evidence of arthropathy [2]. In this report, we describe a similar case with an atypical presentation of FS and the therapies used in the management.

This article was previously presented as a poster at the 2021 Trinitas Research Day Conference on March 31, 2021.

## Case presentation

The patient was a 55-year-old Brazilian woman with no past medical history who presented to the emergency department with complaints of vaginal pain for two weeks. The symptoms had started as mild vaginal swelling and ulcers, which had then progressed to vaginal and lower abdominal pain, intermittent high-grade fever, nausea, and vomiting. Initial vital signs were significant for a temperature of 101.1 ˚F, a pulse of 114/minute, respiratory rate of 18/minute, and blood pressure of 77/43 mmHg. Physical examination showed tenderness in the hypogastric and pelvic regions and whitish clustered vesicular lesions on the left labia minora and fourchette typical of genital herpes. Blood tests revealed a white blood cell (WBC) count of 1,100/mm^3^ (1.1 K/μl) with an absolute neutrophil count (ANC) of 30/mm^3^ (0.03 K/μl).

A sepsis workup consisting of urine, blood, and vaginal cultures, and a CT scan of the abdomen were obtained to evaluate for septic shock. Norepinephrine infusion was initiated as the patient's hemodynamic instability was refractory to intravenous fluid hydration. The patient was started on broad-spectrum antibiotics and valacyclovir and was transferred to the intensive care unit for further management.

The septic workup revealed a diagnosis of genital herpes. Valacyclovir and norepinephrine infusions were continued until the patient was hemodynamically stable. The CT scan of the abdomen showed splenomegaly measuring up to 13.7 cm in the anteroposterior dimension. Given the leukopenia, the patient was empirically started on granulocyte colony-stimulating factor (G-CSF), i.e., filgrastim. The WBC count improved to 6,700/mm^3^ (6.7 K/μl) following five days of treatment with filgrastim. The patient was discharged on oral valacyclovir with an outpatient follow-up appointment at the hematology clinic.

Two weeks following discharge, the patient presented with new-onset right-lower-sided chest pain for three days. The pain was sudden-onset, continuous, sharp in quality, progressive and non-radiating, and was aggravated by deep inspiration. No articular or extra-articular symptoms were reported by the patient. Physical examination was unremarkable except for shallow breathing due to the pain. A chest X-ray was negative for pleural effusion, and hence a provisional diagnosis of pleuritis was made.

The laboratory results showed a WBC count of 1,500/mm^3^ (1.5 K/μl) with an absolute neutrophil count (ANC) of 90/mm^3^ (0.09 K/μl). Hence, the patient was placed on neutropenic precautions and was admitted for further evaluation.

Investigations

Blood work was performed to find out the cause of leukopenia. The complement levels were within normal limits. Serum antinuclear antibodies (ANA) and rheumatoid factor (RF) were positive with anti-cyclic citrullinated peptides (anti-CCP) antibody levels greater than 250 units, thus confirming the diagnosis of RA. X-ray of the feet and hands did not show any erosions or degenerative changes. The anti-double-stranded DNA (anti-dsDNA) antibody test was negative. Inflammatory marker results were significant for erythrocyte sedimentation rate (ESR) of 38 mm/hr and C-reactive protein (CRP) of 6 ng/dl (normal level: <1 ng/dl).

The hematology and oncology team was consulted, and a bone marrow aspiration biopsy was performed (before the initiation of G-CSF therapy), which showed 80-90% hypercellular marrow with trilineage hematopoiesis and myeloid left shift. There was also a 5% clonal T-cell population, positive for clonal T-cell receptor (TCR) gamma gene rearrangement. HTLV1 antibodies, flow cytometry for CD55 and CD59, and antiplatelet antibodies were negative. RBC fragility test and direct and indirect Coombs tests were also negative.

Differential diagnosis

The main differential diagnoses following the bone marrow results were FS or RA-associated T-cell large granular lymphocytic (T-cell LGL) leukemia. Both conditions have analogous clinical and bone marrow biopsy findings as they both result from a similar disease process. However, the absence of marrow lymphocytosis and identifiable LGL cells made T-cell LGL an unlikely diagnosis in this case. 

Treatment

The patient was restarted on filgrastim 480 mcg, with only some improvement in WBC and ANC. Hence, oral prednisone 30 mg twice daily was initiated since the patient had no active infection on this presentation. Significant improvement in WBC count was noted. Filgrastim was subsequently discontinued and the patient was managed with prednisone therapy until discharge (Table 1). Methotrexate was not initiated during the hospitalization as anti-CCP antibody results were not reported initially.

**Table 1:** The patient’s WBC count in response to filgrastim and glucocorticoid treatment by treatment day WBCC: white blood cell count; Abs polys: absolute polymorphonuclear cells; Abs lymphs: absolute lymphocytes; Abs atm: absolute atypical lymphocytes; Hgb: hemoglobin; PltC: platelet count

Day	Medication	WBCC (K/UL)	Abs polys (K/UL)	Abs lymphs (K/UL)	Abs atlym (K/UL)	Hgb (gm/dl)	PltC (K)
1		1.5	0.09	-	0.11	11.8	148
2	Filgrastim	0.7	0.1	0.5	-	8.8	105
3	Filgrastim	0.9	-	0.68	0.04	9.4	93
4	Filgrastim	1.0	-	0.72	-	9.3	82
5	Filgrastim + prednisone 30 mg twice daily	1.4	-	0.87	-	9.0	71
6	Filgrastim + prednisone	1.7	0.03	0.90	0.44	10.3	69
7	Prednisone	5.4	1.46	0.92	0.54	10.7	67
8	Prednisone	16.7	14.1	1.5	-	10.0	75
9	Prednisone	21.5	18.7	1.9	-	10.5	81

Outcome and follow-up

The patient was discharged on a tapering dose of oral prednisone. Thereafter, a dose of 10 mg prednisone was continued. She followed up closely with the medical, rheumatology, and hematology clinics. At the follow-up visits, the WBC and ANC were 1,200/mm^3^ (1.2 K/μl) and 20/mm^3^ (0.02 K/μl), respectively. The dose of prednisone was increased to 20 mg and the patient was started on methotrexate 15 mg weekly. Her latest WBC and ANC are 2,200/mm^3^ (2.2 K/μl) and 70/mm^3^ (0.07 K/μl), respectively (two months following initiation of methotrexate). Since the white cell count was only partially improving on the above-mentioned therapy and the patient started to have some joint pains, the decision was made to initiate a biologic agent.

## Discussion

FS typically presents as a triad of splenomegaly, neutropenia, and erosive arthritis. It is seen in less than 1% of patients with the classic RA [1]. The arthritic changes are usually the first symptoms to manifest and are typically severe and deforming as compared to arthritic changes seen in the classic RA [2]. The arthropathy presents first and worsens over several years before extra-articular symptoms start to develop. In rare cases, FS manifests without arthritic change. Therefore, the diagnosis of FS is based mainly on the presence of persistent neutropenia with an ANC of less than 1500/mm^3^ [2,3]. It is essential to recognize this parameter, as it could be challenging to otherwise diagnose the condition in the absence of arthropathy.

The pathophysiology of FS involves an autoimmune rise in cellular and humoral immune responses. There is an increase in autoantibodies formation against G-CSF and polymorphonuclear neutrophils (PMN), resulting in neutrophil apoptosis and neutropenia [3,4]. Moreover, these patients usually express HLA DR4 - a genetic factor, which is involved in immune complex formation [3]. Additionally, proinflammatory cytokines are activated, which are inhibitory to granulocyte formation [5]. The chronic inflammatory process results in persistent neutropenia. As a result, patients with FS are at an increased risk of recurrent infections. The most common recurrent infections are those of bacterial or fungal origin [6,7].

The treatment of complications (e.g., infection resulting from neutropenia) early on in their presentation is crucial to reducing the mortality rate [7-10]. The long-term management of FS to prevent the incidence of complications is the next step. There are no randomized control trials on the treatment of FS; however, different case reports have highlighted the essential strategies for managing it. The initial choice of therapy depends on the presence of articular disease. Methotrexate should be preferred in patients with articular manifestations. Biologic agents and rituximab are alternative therapies to be used in patients who cannot tolerate or are unresponsive/partially responsive to methotrexate therapy [11,12]. The treatment of neutropenia involves G-CSF. The G-CSF, filgrastim, is indicated when the absolute neutrophil count is less than 100 cells/mm^3^ and the granulocyte count is <1000/mm^3^, due to the increased risk of infections [3]. Several reports show a significant improvement in WBC count within 24 hours following G-CSF initiation in patients presenting with FS-related neutropenia [13,14].

In this patient, the neutropenia improved over five days of G-CSF therapy. However, it was transient, with neutropenia worsening within two weeks after discontinuing treatment. The response was even worse on the second admission (Table 1).

In the absence of an active infection, glucocorticoid is an alternative therapy in patients who do not respond to filgrastim. Glucocorticoid improves neutropenia and, thus, leukopenia by altering granulocyte kinetics through several mechanisms. First, it stimulates the bone marrow to release non-segmented neutrophils into the circulation. In addition, it causes demargination of neutrophils from the endovascular lining while delaying neutrophil apoptosis [15,16]. This results in a significant improvement in WBC count [17]. Due to its immunosuppressive effects, glucocorticoids serve as a bridge to improve WBC counts until patients can be transitioned to other therapies. Our patient responded well to glucocorticoid therapy initially and was later started on methotrexate therapy.

Alternative therapies, including GM-CSF and rituximab, have also been documented in cases of FS-related neutropenia refractory to G-CSF therapy [7,18]. Splenectomy could be considered as a last resort after the failure of the aforementioned therapies [19].

## Conclusions

In the absence of articular disease, diagnosis of FS can be challenging and clinicians should maintain a high index of suspicion. Regarding its management, FS-related neutropenia can be refractory to hematopoietic growth factor therapy like filgrastim and, in the absence of an active infection, glucocorticoid therapy is a superior option in such patients.
